# Attentional Load and Attentional Boost: A Review of Data and Theory

**DOI:** 10.3389/fpsyg.2013.00274

**Published:** 2013-05-20

**Authors:** Khena M. Swallow, Yuhong V. Jiang

**Affiliations:** ^1^Department of Psychology and Center for Cognitive Sciences, University of MinnesotaMinneapolis, MN, USA

**Keywords:** attention, temporal selection, dual-task interference, attentional boost effect, load theory

## Abstract

Both perceptual and cognitive processes are limited in capacity. As a result, attention is selective, prioritizing items and tasks that are important for adaptive behavior. However, a number of recent behavioral and neuroimaging studies suggest that, at least under some circumstances, increasing attention to one task can enhance performance in a second task (e.g., the attentional boost effect). Here we review these findings and suggest a new theoretical framework, the dual-task interaction model, that integrates these findings with current views of attentional selection. To reconcile the attentional boost effect with the effects of attentional load, we suggest that temporal selection results in a temporally specific enhancement across modalities, tasks, and spatial locations. Moreover, the effects of temporal selection may be best observed when the attentional system is optimally tuned to the temporal dynamics of incoming stimuli. Several avenues of research motivated by the dual-task interaction model are then discussed.

Even the earliest writings on attention indicate that it is both limited in capacity and selective in nature (James, 1890; Johnston and Dark, 1986). Since then, extensive controversy has surrounded the nature of those limits and the processing stage at which they occur (Pashler, 1994; Driver, 2001). In all of this, however, few studies challenge the idea that attentional capacity is limited. Increasing attention to one task almost always impairs, or at best has no effect on, performance on a second task (Kinchla, 1992). In contrast to these findings, however, a number of recent reports suggest that transient increases in attention to one task can boost performance in a second encoding task (Lin et al., 2010; Swallow and Jiang, 2010). In this review, we briefly present an influential view of attentional selection (Lavie and Tsal, 1994; Lavie, 2005) that is based on the assumptions that perceptual and cognitive resources are limited. We then review recent findings that challenge these assumptions by demonstrating that increasing attention to one task can sometimes enhance performance in a second task. We propose a new model to account for how a limited-capacity system like attention produces these enhancements.

## Load and Selection

Because attention is limited in capacity (Kinchla, 1992), one must prioritize behaviorally relevant items to ensure that they drive task performance. For decades, attention research has sought to place selective attention within the broader perceptual and cognitive framework (Pashler, 1998). *Early selection* theories (e.g., Broadbent, 1958) suggest that attention acts as a perceptual filter, preventing the identification and semantic analysis of unattended sensory information. *Late selection* theories (e.g., Deutsch and Deutsch, 1963; Duncan, 1980) suggest that selection occurs after sensory stimuli have been identified but before they reach awareness.

The load theory of attentional selection (Lavie and Tsal, 1994; Lavie, 1995; Lavie et al., 2004) reconciled these views by suggesting that attentional selection occurs both early and late in processing, but that early selection varies according to the perceptual and cognitive demands of the attended stimuli. Load theory originated from combining two influential ideas: that attention has limited-capacity (Kahneman, 1973), and that all available perceptual resources will be obligatorily used to process sensory input (Treisman, 1969). This combination leads load theory to two assertions.

First, because perceptual resources are used obligatorily, the upper limit to perceptual processing is also its lower limit. Control processes first direct perceptual resources to goal-relevant (attended) stimuli. Any remaining resources will spill over to irrelevant (unattended) stimuli. As a result, if an attended item (target) requires few perceptual resources to process and identify, then the remaining perceptual resources will “spill over” to process unattended (distractor) stimuli. Late selection then reduces the effect of these irrelevant items on behavior. In contrast, if an attended item requires more perceptual resources, then fewer should spill over to unattended items. Early selection occurs under these circumstances because irrelevant items undergo little perceptual processing. Several factors influence the amount of resources that are needed to perceive an attended item, including the number of distractors (set size), the perceptual similarity between targets and distractors, and stimulus quality (e.g., whether it has been degraded; Lavie and Tsal, 1994; Lavie, 2005).

Evidence for the assertion that excess perceptual resources spill over to distractors came from studies that used the Eriksen flanker paradigm (e.g., Lavie, 1995). Participants indicated which of two-target letters (e.g., a Z or an X) was presented in a central region of the screen. Letters presented in the periphery were task-irrelevant but were associated with a response that was the same as (congruent) or different than (incongruent) the response to the central letter. When the central region contained few letters (low perceptual load), the irrelevant peripheral letter influenced performance, and produced a congruency effect. In contrast, when more letters were present and perceptual load was high, the irrelevant letter’s influence on performance was substantially reduced. This pattern of data has been replicated in studies using other manipulations of perceptual load, including those that increase load by requiring conjunction, rather than feature search, and by degrading the perceptual quality of the stimuli (Lavie, 2005). Moreover, increasing perceptual load decreases the response of brain regions involved in processing task-irrelevant stimuli (e.g., Yi et al., 2004; Bahrami et al., 2007).

A second assertion of load theory accounts for the effects of irrelevant items on task performance (Lavie et al., 2004). Because control processes direct perceptual resources to attended stimuli, any manipulations that impair control processes will disrupt their ability to do so. Therefore, increasing demands on control processes should impair selection, increasing the likelihood that irrelevant items will influence performance. This prediction was confirmed when the low perceptual load condition used in earlier studies was combined with a working memory task (Lavie et al., 2004): The effects of an irrelevant item on task performance were stronger when six items were maintained in memory, rather than one. Importantly, manipulations of cognitive load only affect the processing of irrelevant items when they conflict with relevant items (e.g., both involve spatial processing; de Fockert et al., 2001; Carmel et al., 2012).

Although it is not without controversy (Lavie and Torralbo, 2010; Tsal and Benoni, 2010; Wilson et al., 2011), load theory can account for a large amount of data (Lavie and Tsal, 1994; Lavie, 2005), and encompasses processes that occur throughout task performance. Like other accounts of attention and control, load theory focuses on capacity limitations, both in perception and in control. Here we review evidence that challenges the ubiquity of these limitations, demonstrating that increasing attention to one task can sometimes enhance performance in another task (Lin et al., 2010; Swallow and Jiang, 2010; Swallow et al., 2012).

## Detecting a Target for One Task Broadly Enhances Perceptual Processing

Behaviorally relevant or novel events often signal the need to adapt one’s goals and activities to a new context. In everyday life, such events might constitute a knock at the door, an email notification, or the appearance of a friend one has been waiting for. In the lab, behaviorally relevant events are often pre-defined targets to which participants have been instructed to respond^1^. In all cases, selective attention is needed to identify the stimulus and determine an appropriate response (Chun and Potter, 1995; Nieuwenhuis et al., 2005). Perhaps less obviously, because these events represent a change in the current situation, they may also lead to enhanced perceptual processing of their broader context (Donchin and Coles, 1988; Chun and Jiang, 1998; Bouret and Sara, 2005; Zacks et al., 2007). Consistent with this possibility, extensive data indicate that perceptual and conceptual information that is present when observed activities change form an important component of long-term episodic memory (Newtson and Engquist, 1976; Hanson and Hirst, 1989; Lassiter and Slaw, 1991; Schwan and Garsoffky, 2004; Swallow et al., 2009). However, these data apply almost exclusively to changes in observed activities, rather than to situations in which an event cues an observer to act. Whereas increased attention to context may be expected when activities change, load theory (Lavie, 2005) suggests that increasing attention to a relevant item should decrease the processing of concurrent perceptual information.

The limited-capacity of perceptual processing and attention (Lavie, 2005) necessitates that attending to a relevant event, such as a target, decreases attention to unrelated information that coincides with it. Indeed, most of what is understood about attention predicts that attending to a target should impair, rather than enhance, the processing of concurrently presented but unrelated information. For example, in the attentional blink, participants are typically asked to report the identity of two-target letters that appear in a stream of distractors (Raymond et al., 1992; Chun and Potter, 1995; Dux and Marois, 2009). Items are presented quickly, often at a rate of 10 per second, making their identification difficult. Detecting the first target in the stream reduces the ability to report the identity of the second target when it appears approximately 200–500 ms later. Similarly, in the two-target cost, Duncan (1980) demonstrated that the ability to detect a target is impaired when it coincides with another target, rather than a distractor. Thus, relative to distractor rejection, detecting and responding to a target produces significant demands on attention that reduce the availability of attentional resources for other items.

Over the last several years, however, several studies have presented data that seemingly challenge the ubiquity of interference from target detection. Data from multiple sources, including studies of memory, priming, brain activity, and perceptual learning suggest that attending to a behaviorally relevant target item can actually boost the perceptual processing of concurrent, but unrelated information.

**Figure 1:**
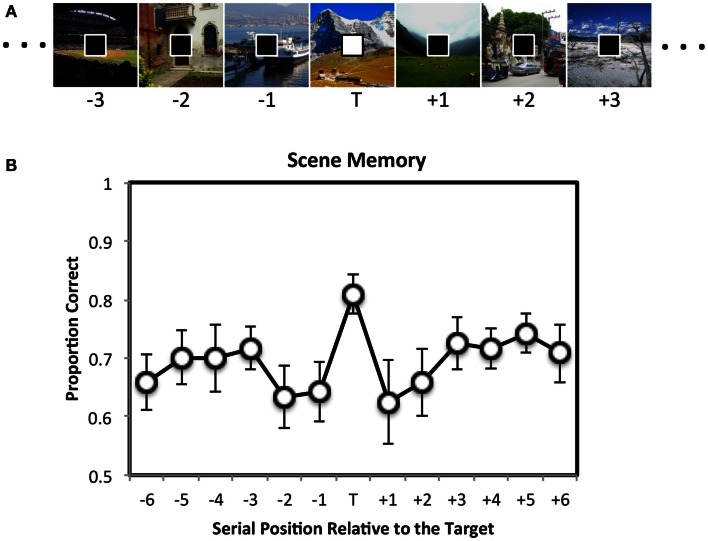
**The attentional boost effect**. **(A)** Participants memorized scenes (500 ms duration, 0 ms ISI) for a later memory test. At the same time, they also pressed a key as quickly as possible whenever the square presented at fixation was white instead of black. Stimuli are not drawn to scale. **(B)** Later recognition memory for the scenes was enhanced if the scene was presented at the same time as a target square during encoding. Error bars = ±1 standard error of the mean. Adapted from Swallow and Jiang (2010).

In one study, Swallow and Jiang (2010) asked participants to perform two continuous tasks at the same time (Figure 1A). For one task participants were shown a series of scenes, one at a time (500 ms/item), at the center of the screen. Participants encoded all of the scenes for a subsequent memory test. For a second task a stream of squares was presented at fixation (also 500 ms/item). The square could be black or white, and participants pressed a key as quickly as possible whenever a white target square appeared. Importantly, the square was completely unrelated to the scene. To examine the effect of target squares on encoding the background scenes, the scenes were assigned to thirteen serial positions around the target square. Scene memory was assessed in a forced choice recognition test at the end of the experiment. If increasing attention to a target leads to widespread increases in perceptual processing, then scenes that are presented at the same time as a target square should be better remembered than those presented with a distractor square^2^ (*enhancement hypothesis*). However, because perceptual and control processes are limited (Lavie, 2005), targets should also reduce the availability of attention for processing the scene. Encoding scenes into memory requires attention (Wolfe et al., 2007). Target detection should therefore interfere with memory for images that coincide with, and even closely follow a target (*interference hypothesis*). This, however, did not occur. Instead, memory for scenes that were presented at the same time as a target square was enhanced relative to those presented with a distractor square (Figure 1B). No consistent differences were observed in memory for scenes that appeared with a distractor in the other serial positions. These data suggest that perceptual processing increases when behaviorally relevant events occur, resulting in a global enhancement of multiple competing tasks.

Importantly, this pattern of data could not be attributed to the perceptual salience of the rare, white square (Swallow and Jiang, 2010). No memory advantage was observed for scenes that were presented at the same time as a white square when the squares were ignored. In addition, the effect was not due to a motor response, as it also occurred when participants were asked to covertly count the number of target squares (Swallow and Jiang, 2012). Although detecting the target square required more attention than rejecting a distractor square (Duncan, 1980; Raymond et al., 1992), increasing attention to the square task boosted performance in the second task – an *attentional boost effect* (Swallow and Jiang, 2010)^3^.

The attentional boost effect is not limited to tasks that require participants to actively encode stimuli for a later memory test. In an experiment examining implicit memory (Spataro et al., 2013), participants read aloud words that were individually presented at a rate of 2 per second. Each time a word appeared a green or red circle appeared below it. In the divided attention condition, participants pressed a button when the circle was green. In the full attention condition, they ignored the circle. After completing the encoding task and a brief delay, participants performed a lexical decision task on exposed and unexposed words. Remarkably, words that coincided with targets produced nearly twice as much priming as words that coincided with distractors. Moreover, this advantage was absolute: priming was greater for words presented with targets than for words in the full attention condition. This pattern of data was replicated in a word fragment completion task. It did not, however, occur in a conceptual priming task, suggesting that target detection enhanced the perceptual encoding of concurrently presented words.

The effects of detecting a target on concurrent image processing can also be observed in short-term memory tasks. In their study, Lin et al. (2010) first familiarized participants with scenes. In a subsequent task, 16 familiar scenes were presented one at a time (133 ms duration, 367 ms ISI) on each trial. A letter was presented in the center of each scene, and participants reported the identity of the gray letter at the end of each trial. They were also shown a scene and asked to indicate whether it was presented during the trial. Thus, this and similar experiments (e.g., Leclercq and Seitz, 2012a) examined how detecting a target letter influenced memory for whether a familiar image was recently presented. Consistent with the effects of targets on long-term visual memory, target detection enhanced short-term source memory for scenes.

Target detection also enhances short-term memory for semantically impoverished stimuli (Makovski et al., 2011). Participants performed a change detection task on color arrays separated by a 1500 ms delay. A letter was presented at fixation and participants quickly pressed a button when the letter was a T. The letter could appear at the same time as the first color array, during the 1500 ms retention interval, or at the same time as the second color array. Participants were better able to detect a color change when a target letter was presented than when a distractor letter was presented. Importantly, this benefit occurred only when the target letter coincided with the first color array, suggesting that target detection facilitated the encoding of the color patches into memory, but not their retention or comparison to current perceptual input. Interestingly, these data might help account for an earlier report of enhanced change detection in scenes when targets are present (Beck et al., 2001). Although no statistical analyses were reported, performance on the change detection task was better when a target letter was present (41%) than when it was absent (51%). These data offer initial evidence that the selection of behaviorally relevant events enhances the encoding of information into short-term memory.

**Figure 2:**
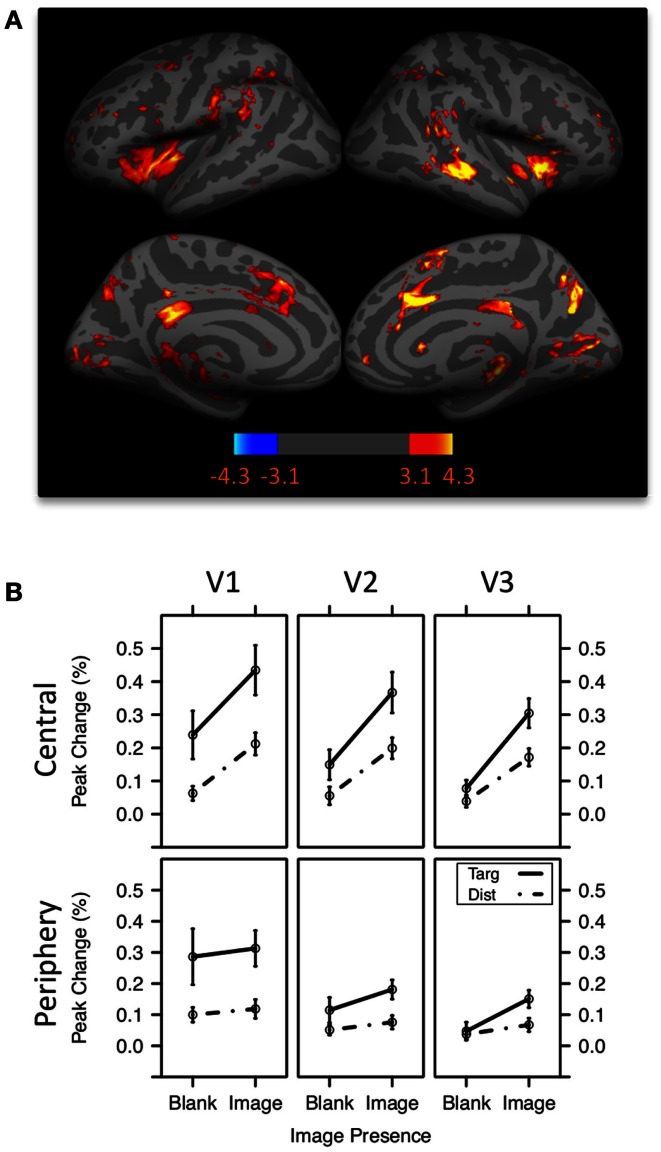
**The target-mediated boost**. **(A)** Target tones were associated with increased activity in a network of brain regions previously associated with attentional selection. Color bar values indicate z statistics for the target-distractor contrast. **(B)** Peak percent change in activity in retinotopically defined early visual areas V1, V2, and V3 representing the central and peripheral visual fields following tones. V1 increased more in activity following the presentation of a target tone than a distractor tone (indicated by the difference between the solid and dashed lines). The effect was present in both central and periphery regions, but diminished in magnitude from V1 to V3. Error bars represent ±1 standard error of the mean. Adapted from Swallow et al. (2012).

Other evidence that target detection produces broad encoding enhancements comes from a recent fMRI study (Swallow et al., 2012). Participants pressed a button as quickly as possible whenever a tone of a pre-defined pitch was presented over headphones. If increasing attention to an auditory target pulls perceptual resources away from visual regions of the brain (Shomstein and Yantis, 2004; Johnson and Zatorre, 2006), then activity in visual areas should decrease when an auditory target is presented. If, however, temporal selective attention leads to widespread perceptual enhancements, then activity in visual areas should increase more when an auditory target is presented, rather than a distractor. The data confirmed the latter prediction. Activity in early visual cortex increased when an auditory target was presented, rather than a distractor (Figure 2). These data indicate that the response of early visual areas to goal-relevant events (Jack et al., 2006) is mediated by attention. In addition, unlike spatial selective attention (e.g., Kastner et al., 1998; Silver et al., 2007), temporal selection of an auditory target produced effects that were not spatially localized and that decreased in magnitude from early to late visual areas. This effect was present when auditory tones were presented on their own and when they were presented at the same time as a face, scene, or scrambled image. Moreover, the same pattern occurred when visual targets were presented with visual scenes. Under these conditions, detecting a target in the central visual field led to enhanced activity in regions representing the visual periphery and in early auditory cortex. These data rule out the possibility that the increase in early visual cortical activity in response to target tones reflects purely multi-modal processing in a region that is traditionally considered unisensory (Brosch et al., 2005; Baier et al., 2006; Ghazanfar and Schroeder, 2006; Driver and Noesselt, 2008; Kayser et al., 2008). Rather, temporal selection of a target, but not distractor rejection, boosts activity in perceptual regions of the brain that are not involved in its processing (*target-mediated boost*).

The effects of target detection on perceptual processing are not limited to tasks involving visual stimuli, or to situations in which the background image and the target overlap in space. As just reviewed, the target-mediated boost is observed even in a purely auditory task (Swallow et al., 2012). Furthermore, both long-term and short-term memory for scenes is enhanced when they coincide with the presentation of an auditory target, such as a high-pitched tone presented in a stream of low-pitched tones (Lin et al., 2010; Swallow and Jiang, 2010). Spatial overlap is also unnecessary. Short-term memory for color patches presented several degrees from fixation is enhanced by the presence of a target letter during encoding (Makovski et al., 2011), and scenes presented in an unattended location also benefit from target detection (Leclercq and Seitz, 2012a). Combined, these data suggest that target detection produces enhancements that are not specific to the spatial location or modality of the target.

Finally, perceptual learning data further support the claim that target detection results in widespread perceptual enhancements (Watanabe et al., 2001; Seitz and Watanabe, 2003). For these studies, participants identified gray letters in a stream of black letters. Each letter was presented in the center of an irrelevant random dot motion display (RDM). Motion coherence in these displays was below threshold, so learning was unconscious. Importantly, one direction was always paired with the gray target letters. Following nearly 20,000 trials, perceptual learning was obtained only for the direction of motion paired with the target letter, but not for motion directions paired with a distractor letter. Detecting the target letter increased sensitivity to concurrently presented, task-irrelevant, and unattended, perceptual information (Seitz and Watanabe, 2003). Interestingly, *task-irrelevant perceptual learning* (TIPL) is strongest for motion features processed in primary visual cortex (V1) and located near the target (Watanabe et al., 2002; Nishina et al., 2007). TIPL is clear evidence that behaviorally relevant events can influence context processing. However, it is slow to develop and restricted entirely to information that slips past attentional filters. In fact, no learning occurs when participants are able to detect the dominant direction of motion in the RDM displays and presumably suppress it (Tsushima et al., 2008; see also Dewald et al., 2011). Although there are similarities between TIPL and the attentional boost effect, inconsistencies such as these require further investigation.

Together these data indicate that selectively attending to behaviorally relevant events can enhance the processing of, and memory for, concurrently presented information. These effects are immediate and long lasting, influencing activity in perceptual regions of the brain (Swallow et al., 2012), short-term memory for color arrays and scenes (Lin et al., 2010; Makovski et al., 2011), long-term memory for visual stimuli (Swallow and Jiang, 2010), implicit memory for words (Spataro et al., 2013), and perceptual sensitivity to orientations and directions of motion (Seitz and Watanabe, 2003; Seitz et al., 2009). The fact that many of these effects occur cross-modally suggests that detecting goal-relevant events such as a target has broad effects on perceptual processing.

The attentional boost effect can be distinguished from previous demonstrations of enhancements that occur across tasks. Previous observations that two tasks and stimuli can interact have been limited to situations in which the tasks and items are semantically congruent. For example, masked images (e.g., a dog) are more easily identified when they are presented at the same time as a semantically congruent sound (e.g., barking), rather than an incongruent sound (e.g., hammering; Chen and Spence, 2010; see also Griffin, 2004). Furthermore, holding a word or image in memory increases the likelihood that semantically congruent stimuli will be attended (Soto and Humphreys, 2007). In contrast to these findings, the attentional boost effect is unique in demonstrating that cross-task enhancements can occur for stimuli that are unrelated but concurrently presented. The targets and distractors are completely unrelated to the background images.

## Temporal Selection Drives the Attentional Boost Effect

The experiments just reviewed point to a robust and broad processing advantage for information that coincides with targets. These data contradict the near ubiquitous finding that increasing attention to one task impairs performance on another (Kinchla, 1992). The availability of attentional resources appears to vary rapidly over time and is greater in some moments (when targets are detected) than in others. This fluctuation creates difficulties for limited-capacity theories such as the load theory. As a result, it is of critical importance to address whether alternative explanations can account for the attentional boost effect.

An immediate concern is that detecting a target may not have required more attention than did rejecting a distractor. Although target detection demands attention (Duncan, 1980; Chun and Potter, 1995), it is possible that the target square was too easily distinguished from the distractor squares and did not sufficiently tax perceptual resources. To address this concern, in one study we changed the simple color-detection task to a task that involved conjunction search (Swallow and Jiang, 2010). For this task, participants pressed a button for a target letter (e.g., a Red-X) that differed from distractor letters (e.g., Red-Y’s and Blue-X’s) in the combination of color and shape. Under these conditions, the target was perceptually similar to distractors, so perceptual load should have been high (Lavie and Tsal, 1994). In addition, distinguishing targets from distractors when they are defined by the conjunction of two features requires selective attention (Treisman and Gelade, 1980). The attentional boost effect was found under these conditions, indicating that it occurs even when targets are difficult to distinguish from distractors.

Another class of potential explanations for the attentional boost effect stem from the possibility that it reflects attentional phenomena that have already been well characterized in the literature. In particular, targets may have alerted participants and increased arousal, effectively increasing the amount of attention available to perform the two tasks (Posner and Boies, 1971). However, an inspection of Figure 1B makes it clear that there was no memory advantage for scenes that were presented immediately after the target, when the effects of alerting and arousal should have been greatest. Memory for scenes that followed a target was no better than memory for scenes that preceded it (Swallow and Jiang, 2010, 2011). Moreover, temporal selective attention produces a pattern of brain activity in early visual cortex that is distinct from the effects of alerting and arousal. Unlike alerting, detecting an auditory or visual target increases activity more strongly in primary visual cortex (Swallow et al., 2012), than in late visual areas (Anderson et al., 2003; Thiel et al., 2004; Fan et al., 2005).

**Figure 3:**
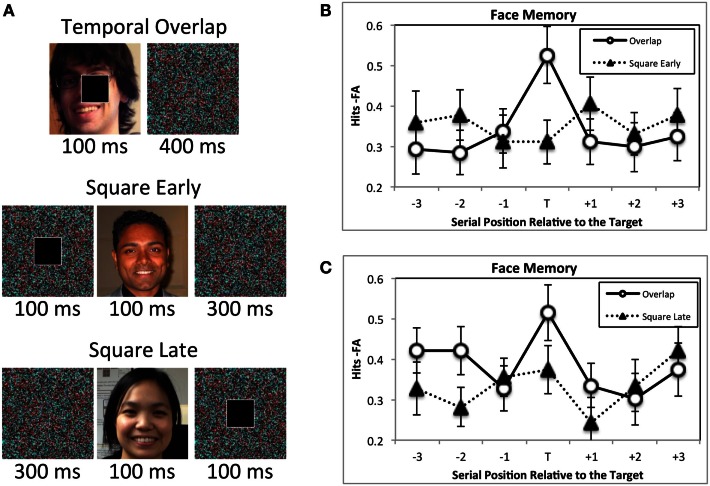
**The attentional boost effect occurs only for images that coincide with a target in time**. **(A)** In two experiments participants were asked to memorize faces (100 ms duration, 400 ms mask; faces used in the experiment were famous), and to press a button when a white square, rather than a black square appeared (square duration = 100 ms). For one experiment the square and face onset at the same time in some blocks of trials (Temporal Overlap condition). In the other blocks of trials the square onset 100 ms before the face onset (Square Early condition). In the second experiment, temporal overlap blocks were interspersed with blocks in which the square onset 100 ms after the face (Square Late condition). **(B,C)** Target detection enhanced memory for faces only when the target and face overlapped in time. It did not facilitate memory for images that occurred 100 ms earlier **(B)** or 100 ms later **(A)**. Error bars represent ±1 standard error of the mean. Adapted from Swallow and Jiang (2011).

Another possibility is that the target could have cued attention to the background scene. Attentional orienting in response to a cue has its largest effects 100–200 ms later (Nakayama and MacKeben, 1989; Egeth and Yantis, 1997). If the target acts as an attentional cue, then images that are presented during this brief time window should be better encoded into memory than those presented at the same time as a target. However, this is not the case. In one experiment (Swallow and Jiang, 2011) faces were presented for 100 ms and then masked for 400 ms (Figure 3). In different blocks of trials the target and distractor squares either onset at the same time as the face, or onset over the mask 100 ms before the face was presented. A memory advantage was observed only for faces that onset at the same time as the target. Moreover, another experiment found no evidence of enhanced memory for a face when it preceded a target (Swallow and Jiang, 2011), suggesting that the effects of target detection are temporally constrained.

Alternatively, it is possible that the effects of target detection on learning and memory are due to their distinctiveness. Items that are semantically or perceptually distinct from other items in a study list are better remembered than those that are not (Schmidt, 1991; Fabiani and Donchin, 1995; Hunt, 1995). However, recent data indicate that the attentional boost effect in short- and long-term memory is just as strong when target squares are as common as distractors (a 1:1 target to distractor ratio) and when they are relatively rare (a 1:6 ratio; Makovski et al., 2011; Swallow and Jiang, 2012). The target-mediated boost in fMRI is also observed when targets and distractors are equally frequent (Swallow et al., 2012). Moreover, poorer memory is observed for images that coincide with infrequent distractors rather than with distractors that are common (Swallow and Jiang, 2012). Distinctiveness is neither necessary nor sufficient for the attentional boost effect.

A final consideration is the nature of the attentional boost effect itself. Rather than an enhancement due to target detection, the attentional boost effect could reflect poorer memory for images presented with distractors. Several lines of evidence argue against this possibility. First, TIPL represents an increase in sensitivity for visual features that coincide with a target following training, and no change in sensitivity for those that coincide with distractors (Seitz and Watanabe, 2009). Second, in a study examining short-term memory for familiar scenes, scene memory was significantly above chance only when it was paired with a target, but not when the scene appeared on its own or with a distractor (Lin et al., 2010). Third, when task demands were held constant, long-term memory for faces that were presented at the same time as a distractor was similar to that for faces that were presented on their own (Figure 3; Swallow and Jiang, 2011). Finally, priming is enhanced for words presented with a target circle and unaffected for words presented with a distractor circle, relative to a condition in which the circles were task-irrelevant (Spataro et al., 2013). It therefore appears that the relative advantage for visual information that coincides with a target, rather than a distractor, reflects an enhancement due to target detection.

## Reconciling the Attentional Boost with Load

The available data support the contention that, despite requiring attention, detecting a target can boost the processing of concurrently presented information. This finding challenges the notion that all perceptual resources are used obligatorily (Lavie and Tsal, 1994): If perceptual processing broadly increases at some moments in time (e.g., when targets are detected), then it may not have been fully used at other moments in time. The attentional boost effect also represents a significant challenge to the long held view that performance in one task will suffer when another task or item requires more attention. If temporal selective attention of one target impairs the ability to detect a second target that is presented at the same time (Duncan, 1980), or soon after (Raymond et al., 1992), then how does it also enhance the encoding of concurrently presented perceptual information?

This section focuses on accounting for the potential effects of target detection on stimulus encoding. We propose that the encoding enhancement that is captured in the attentional boost effect and related phenomena represents a previously unrecognized feature of temporal selective attention that operates alongside dual-task interference.

**Figure 4:**
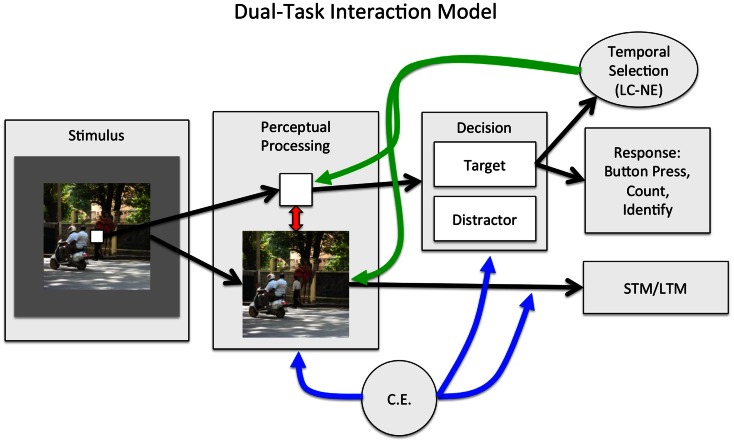
**The dual-task interaction model**. On each trial, sensory information from the two task-relevant stimuli is selected to undergo perceptual processing (early selection), as directed by the central executive (CE). Within perceptual processing areas, the two stimuli compete for representation, producing dual-task interference. Dual-task interference also arises from the need to maintain multiple goals simultaneously: intentionally encoding the scene into memory, and generating an appropriate response to the square. Perceptual evidence that the square is a target or distractor is accumulated, and the square is categorized once a threshold has been reached. The item may then be selected to guide behavior and be maintained in memory if necessary (late selection). The categorization of the square as an item that requires a response (e.g., counting, holding on to the identity of the item in memory, or generating, or withholding, a planned motor response) triggers temporal selective attention. We propose that temporal selective attention, potentially instantiated by a transient increase in the release of norepinephrine from the locus coeruleus (LC-NE; Nieuwenhuis et al., 2005), is temporally, but not modality or spatially selective. It therefore enhances the processing of all perceptual information that is present when a target occurs. In addition, the detection of a target could reset neuronal activity across regions (Bouret and Sara, 2005; Lakatos et al., 2009). When stimuli are regularly presented, the consequent entrainment of neural activity to those stimuli could increase the efficiency of task processing.

Although many questions about the attentional boost effect remain, the available data provide a basis for proposing an extension to what is currently understood about temporal attention and selection. As in most models of attentional selection (e.g., Lavie and Tsal, 1994; Desimone and Duncan, 1995), the *dual-task interaction model* (Figure 4) proposes that task goals, maintained by a cognitive control mechanism like the central executive (Baddeley, 2003), prioritize the perceptual processing of goal-relevant stimuli. Goal-based attentional prioritization occurs early in perception, ensuring that relevant stimuli are perceptually processed. It also occurs post-perceptually, ensuring that those stimuli are maintained in memory if necessary and lead to task-appropriate responses. The dual-task interaction model is entirely consistent with load theory’s claims that selection occurs at multiple stages, and that cognitive control plays a critical role in ensuring that relevant information is used to guide task performance (Lavie, 2005).

The dual-task interaction model extends load theory and other theories of dual-task performance with two components. The first is a broad attentional enhancement that is triggered by the appearance of a target in a stream of distractors. This enhancement roughly corresponds to temporal selective attention mechanisms described by others (e.g., Bowman and Wyble, 2007; Olivers and Meeter, 2008) and is closest conceptually to a model of the attentional blink that is based on the locus coeruleus–norepinephrine (LC-NE) system (Nieuwenhuis et al., 2005). However, the dual-task interaction model emphasizes the broad and spatially unconstrained perceptual enhancements that result from temporal selection. The second component is the coupling of task processes when stimuli are rhythmically presented. Although we propose that detecting a target always triggers an attentional enhancement, this effect may be more easily observed when the stimuli are rhythmically presented. Rhythmic stimulus presentation promotes efficient processing (Jones et al., 2002; Schroeder and Lakatos, 2009; Mathewson et al., 2010) and the temporal coupling of task processes.

### Temporal selection broadly enhances perceptual processing

Within the dual-task interaction model, the decision that an item is a target leads to response selection and production, which are determined by the current goal set. It also leads to temporal selection, which enhances perceptual processing (Figure 4). To account for the finding that detecting a target results in the enhanced processing of the target and its context, the dual-task interaction model proposes that temporal selection is selective for time, but not for space or modality.

The target-mediated boost, makes it clear that temporal selective attention is not simply the brief application of spatial selective mechanisms (Swallow et al., 2012) (although the effects of both types of selection are likely to overlap and could interact; Coull and Nobre, 1998; Nishina et al., 2007; Leclercq and Seitz, 2012a). Indeed, the challenges facing temporal selection are distinct from those facing spatial selection. Rather than resolving competition in neural receptive fields (Desimone and Duncan, 1995), temporal selection must ensure that sufficient information is acquired about relevant items and their context before their processing is disrupted by new input. One way temporal selection may ensure that information about such items is available for task performance is to prioritize it for maintenance in short-term memory (e.g., Chun and Potter, 1995). However, perceptual processing takes time (Schyns and Oliva, 1994; Ploran et al., 2007) and encoding can be easily disrupted by new input (Breitmeyer and Ganz, 1976; Potter et al., 2004). Temporal selection therefore may also enhance perceptual processing to ensure that information about the relevant item and its context is encoded into memory. Without such an enhancement, perceptual information about behaviorally relevant items and their context could be lost.

The notion that temporal selection ensures that goal-relevant information is available to influence task performance may be best captured by theories that account for the attentional blink. Although they differ in their particulars, most theories of the attentional blink suggest that it reflects the protection of high-level representations of the target from interference (Dux and Marois, 2009). For example, in the Boost and Bounce Theory of temporal attention (Olivers and Meeter, 2008), the recognition of a target triggers an excitatory feedback response to perceptual areas, beginning with those that represent item identity. This response enhances, or boosts, the likelihood that a goal-relevant item will be maintained in working memory and, consequently, influence behavior. To avoid enhancing items that could interfere with task performance, the recognition of a distractor item results in inhibitory feedback to these same areas, reducing the likelihood that subsequent items will reach awareness. Similarly, the simultaneous type, serial token model (ST^2^) proposed by Bowman and Wyble (2007) claims that the classification of an item as a target triggers an attentional “blaster.” This blaster allows the features of the target item to be bound into an episodic and individuated representation that is actively maintained in memory until it is needed. In the ST^2^ model, the enhancement is automatically followed by inhibition. In both of these models, the mechanism that produces the attentional blink most closely corresponds to late selection, as its primary function is to determine which stimuli reach awareness and working memory (Vogel et al., 1998), rather than to prevent the perceptual processing of task-irrelevant information.

Like most theories of temporal attention, these two theories focus on explaining how temporal attention protects a target item from interference at the same time that it suppresses the processing of items that soon follow it (Dux and Marois, 2009). Like load theory (Lavie and Tsal, 1994; Lavie et al., 2004) however, their focus is almost exclusively on how a single relevant item is prioritized. In contrast, the dual-task interaction model proposes that temporal selection also enhances perceptual processing in regions that are not involved in representing the target. Although it is not an explicit component of most theories of temporal selection, the LC-NE model (Nieuwenhuis et al., 2005) does suggest that the effects of temporal attention may in fact be widespread. The LC-NE account of the attentional blink proposes that it reflects the dynamics of the LC-NE response to targets. In monkeys, a behavioral response to targets is reliably preceded by a phasic increase in the release of norepinephrine from the LC (Aston-Jones et al., 1994). NE increases the responsivity of target neurons to their input (Servan-Schreiber et al., 1990; Aston-Jones and Cohen, 2005). As a result, it could provide the neurophysiological basis for temporal selection as well as the attentional blink (Aston-Jones and Cohen, 2005; Nieuwenhuis et al., 2005). Of importance to the dual-task interaction model, the LC projects widely throughout neocortex. The effects of the phasic LC-NE response to targets therefore are likely to be widespread, spanning different sensory modalities and representing different spatial locations (Aston-Jones and Cohen, 2005).

The neurophysiological mechanisms that underlie the attentional boost effect and related phenomena are unknown. However, the broad perceptual enhancements that result from target detection (Seitz and Watanabe, 2009; Lin et al., 2010; Swallow and Jiang, 2010, 2011; Makovski et al., 2012; Swallow et al., 2012; Spataro et al., 2013) are a plausible consequence of phasic LC-NE signaling.

One potential effect of temporal selective attention on neural processing could be to reset the phase of neural oscillations in a diverse network of cortical areas (Schroeder and Lakatos, 2009). This, combined with work suggesting that the phasic LC-NE response to goal-relevant events can reset neuronal activity (Bouret and Sara, 2005) reinforces the proposal that the effects of targets on neural activity are widespread. They may also provide an explanation for one of the more surprising aspects of the target-mediated boost (Swallow et al., 2012): Detecting an auditory tone increases activity in early visual areas, even when no visual stimuli were presented. It is possible that these data reflect the resetting of neuronal activity in these areas, modulating their sensitivity to new input (Lakatos et al., 2009). The next section discusses the consequences of phase resets in greater detail.

As with the phasic LC-NE response to targets (Aston-Jones and Cohen, 2005), we propose that the perceptual enhancements resulting from temporal selection occur whenever a target is detected. However, the ability to detect these enhancements is likely to be a function of many different factors. One factor is the presence of interference effects later in processing. Performance of even the simplest tasks involves multiple mechanisms, some that are parallel (e.g., perception) and some that are serial (e.g., response selection; Pashler, 1994). Although two stimulus streams may be perceptually processed in parallel, their encoding into working memory, and the generation of appropriate responses are likely to be limited by serial mechanisms (Pashler, 1994). Therefore, enhancements in perceptual processing may not translate into better performance when the consolidation or maintenance of perceptual information in long-term and short-term memory is disrupted. Indeed, increasing the difficulty of response selection by asking participants to make different, arbitrary responses to different targets eliminates (but does not reverse) the memory advantage for scenes presented at the same time as targets (Swallow and Jiang, 2010). Load theory (Lavie et al., 2004) also suggests that increasing cognitive load might interfere with the ability to observe the broad effects of temporal selection. Reducing the availability of cognitive resources to maintain or consolidate perceptual information into memory should reduce the utility of perceptual processing enhancements produced by temporal selection.

### Rhythmic stimuli promote the coupling of task processes

A second component of the dual-task interaction model is the proposal that the temporal structure of the stimulus streams may play a critical role in how much temporal selection for one task influences performance in another. Attentional boost effect experiments that irregularly presented task stimuli tended to show a weaker memory advantage for information that coincided with targets than experiments with regularly presented stimuli (e.g., 3–5% effects in Makovski et al., 2011 and Swallow et al., 2012 vs. 10–20% effects in Swallow and Jiang, 2010, 2011, 2012). This difference across studies could be explained by recent research that examines how rhythmic stimuli influence one’s attentional state. Visual neural activity can entrain both to rhythmically presented stimuli and to activity in other sensory areas, enhancing the effects of temporal selection and integrating information across modalities (Large and Jones, 1999; Jones et al., 2002; Lakatos et al., 2007, 2008; Schroeder and Lakatos, 2009; Busch and VanRullen, 2010; Mathewson et al., 2010).

Neuronal oscillations correlate with how easily input can drive the activity of neural populations. In one influential study, Lakatos et al. (2008) trained monkeys to attend to either visual or auditory stimuli that were alternately presented in a continuous stream. Occasionally an oddball stimulus was presented, signaling the monkey to make a motor response. Two findings that are particularly relevant to the attentional boost effect were reported. First, activity in supragranular layers of visual cortex entrained to attended stimuli, regardless of whether those stimuli were presented in the auditory or visual modality. This entrainment could reflect the phase resetting of activity in visual cortex in response to an attended event (Lakatos et al., 2007). The second relevant finding was that the speed with which the monkeys responded to an oddball stimulus was influenced by when it occurred relative to the phase of low frequency (delta) neuronal oscillations. Faster responses were generated to stimuli presented when the neurons were most excitable (Lakatos et al., 2008; Schroeder and Lakatos, 2009).

Oscillatory activity in EEG recordings also appear to influence attention and perception in humans (Mathewson et al., 2009, 2010; Busch and VanRullen, 2010). Visual stimuli are more easily detected when they are presented at the peak of an alpha wave in EEG recordings (Mathewson et al., 2009). Moreover, behavioral data further indicate that the entrainment of cortical activity across visual and auditory regions has widespread effects on attention. Attention to an item is enhanced when it occurs at a moment in time that is predicted by the rhythm of stimuli that precede it, regardless of whether they were in the same or different modalities (Klein and Jones, 1996; Jones et al., 2002; Miller et al., 2013). It therefore appears that attention to rhythmic stimuli can encourage synchronous activity across a network of cortical areas (including those involved in higher-order cognitive processes, Besle et al., 2011), which in turn makes them maximally sensitive to input at similar points in time.

This possibility is captured in the proposal that attention to perceptual information operates in two different modes (Schroeder and Lakatos, 2009). In the *rhythmic* mode perceptual regions of the brain are maximally sensitive to input at the moment in time that input is expected (see also Large and Jones, 1999; Baier et al., 2006). The rhythmic mode is therefore advantageous when stimuli are presented in simple regular sequences. However, it comes with the cost of introducing long periods of time in which perceptual regions are less responsive to their input; periods of high excitability are interspersed with periods of low excitability. If stimuli appear irregularly or in isolation, then adopting a rhythmic processing mode could be maladaptive. In these situations, attention may shift into what Schroeder and Lakatos (2009) refer to as the *continuous* mode of processing. This processing mode is less efficient, but is also better able to maintain neural excitability over long periods of time.

In the dual-task interaction model we propose that the regular presentation of stimuli for both tasks encourages the adoption of a rhythmic processing mode. This, in turn, allows for greater apparent interaction between areas and processes that are involved in performing the detection task and the encoding task. In this situation, the broad effects of temporal selective attention may more efficiently influence multiple tasks and stimuli when regions involved in performing them are optimally excitable at the same time. As a result, the effects of temporal selection should be more easily detected when stimuli are presented regularly, rather than irregularly. In the latter condition, the attentional boost effect may be small and more difficult to detect.

### Load theory and the dual-task interaction model

As reviewed previously, load theory (Lavie, 2005) proposes that limits in perceptual and cognitive processing are accommodated by both early and late selection mechanisms. Early selection ensures that perceptual resources are directed to goal-relevant items. Late selection ensures that goal-relevant items reach awareness and influence behavior once they have been perceptually processed. To account for the late selection data, load theory asserts that all perceptual resources are used: attended items are processed, but any perceptual capacity that remains spills over to irrelevant items (Lavie and Tsal, 1994).

The dual-task interaction model does not contradict the claim that selection can happen both early and late in processing. It is also consistent with dual-task interference and limitations in post-perceptual processing more generally (Pashler, 1994). According to the dual-task interaction model, responding to a target should increase demands on control processes. However, a corresponding reduction in control resources devoted to the encoding task can be offset by enhancements to perceptual processing that result from temporal selection. Thus, the dual-task interaction model reconciles the attentional boost effect with several aspects of load theory and the broader dual-task interference literature (e.g., Kinchla, 1992; Pashler, 1994).

The dual-task interaction model’s suggestion that perceptual processing varies as a function of temporal selection, however, is difficult to reconcile with load theory’s claim that all perceptual resources are obligatorily used (Lavie and Tsal, 1994). Although alerting and arousal are thought to influence the amount of available perceptual resources (Lavie and Tsal, 1994), the attentional boost effect conforms to neither of these (Swallow and Jiang, 2012; Swallow et al., 2012). In fact, the attentional boost effect lasts no more than 100 ms and is constrained to information presented concurrently with, rather than after, a target (Swallow and Jiang, 2011). If all available perceptual resources are used all the time, then it is not clear how such short-term variability in perceptual processing would occur, even in dual-task situations.

These inconsistencies suggest several possibilities. One is that this aspect of load theory is wrong – perceptual resources can be held in a reserve that is tapped when goal-relevant items appear. However, one could argue that we are comparing apples to oranges. Perceptual load theory describes attentional selection in space. In addition, whereas dual-task interference is important for explaining the effects of cognitive load on spatial selection, the effects of perceptual load can be observed in single tasks (Lavie, 2005). In contrast, the attentional boost effect involves selection over time and is usually observed in dual-task situations. However, the effects of target detection on early visual cortical activity occur even in single task situations (Swallow et al., 2012). Although one could argue that load theory accurately describes spatial selection processes, adhering to load theory’s claim that all perceptual resources are used requires asserting that perceptual capacity rapidly increases when task-relevant events occur. It is not clear how such a claim could be falsified.

Another possibility is that the dual-task interaction model is wrong, and that temporal selective attention of a target item does not broadly enhance perceptual processing. Enhanced memory for a scene that coincides with a target could reflect post-perceptual effects of temporal selection. However, the data strongly suggest that target detection influences perceptual processing, even if it also influences post-perceptual processing. Target detection increases activity in early perceptual areas that are uninvolved in target processing (Swallow et al., 2012), enhances perceptual, but not conceptual priming (Spataro et al., 2013), and facilitates perceptual learning (Seitz and Watanabe, 2003). Although additional research is needed to clarify which stages of processing temporal selective attention enhances, the evidence points to perception.

A final possibility is that broad enhancements in perceptual processing produce unrecognized costs. Most studies of the attentional boost effect use recognition tests that do not capture differences in memory for perceptual details. Image encoding takes place at multiple scales, with coarser, more conceptual information extracted more rapidly than fine-grained perceptual details (Schyns and Oliva, 1994). Although memory for scene orientation was examined in one study (Swallow and Jiang, 2010), the data were noisy and inconclusive. Future research will need to determine whether temporal selection broadly enhances the processing of fine, as well as coarse, perceptual information.

## Implications and Open Questions

In its current form, the dual-task interaction model represents an initial attempt to account for the facilitatory effects of target detection on a concurrent encoding task, despite the increased demands on attention. Like the attentional boost effect itself, this model raises questions about the nature of temporal selective attention, its spatial characteristics, and the roles that load, reinforcement learning, and different attentional states may play in the ability to perform multiple tasks at once.

The dual-task interaction model proposes that temporal selection is broad and not constrained to particular locations or modalities. Although this claim is consistent with the available data, there is only one published study that attempts to address the spatial distribution of the effect (Leclercq and Seitz, 2012a). Additional research investigating both the spatial distribution and time course of temporal selective attention is needed. Moreover, the degree to which these effects are modulated by spatial attention and the relevance of the background information is also unclear. Most studies that have shown an effect of target detection on memory, rather than on perceptual learning, have done so by asking participants to attend to the background images (e.g., Lin et al., 2010; Swallow and Jiang, 2010; Spataro et al., 2013). In one study that examined incidental memory for the background scenes, no advantage for the scenes presented with targets was observed (Swallow and Jiang, 2011). Another recent study found that making targets difficult to perceive may eliminate the memory advantage for concurrently presented scenes (Huang and Watanabe, 2012). Along these lines, it will be important for future research to better characterize how different types of load influence the attentional boost effect. In its current form, the dual-task interaction model suggests that perceptual load and cognitive load may have very different effects on the ability of temporal selection to enhance perceptual processing. A better understanding of how attention and task relevance influence the attentional boost effect will be critical for the development of the dual-task interaction model and its reconciliation with load theory.

The close correspondence between the attentional boost effect and TIPL raises the question of whether they reflect the same mechanism operating on different time scales (Leclercq and Seitz, 2012a). In this and other papers we have proposed that this mechanism is temporal selection. However, TIPL has been explained by appealing to reinforcement learning in the attention-gated reinforcement learning model (AGREL; Seitz and Watanabe, 2009; Roelfsema et al., 2010). According to this perspective, detecting a target is intrinsically rewarding, and therefore triggers the release of neuromodulators that reinforce neural activity in perceptual areas. As a result, the visual system slowly becomes more sensitive to perceptual features that are present when a target occurs. This is consistent with the finding that external rewards, such as the delivery of water, also produce similar perceptual learning effects (Seitz et al., 2009). The dual-task interaction model, in contrast, suggests that the effects that are captured in short- and long-term memory reflect temporal selection rather than reinforcement learning. Although similar, the dual-task interaction and AGREL models differ in what they propose is happening in perceptual areas. Whereas the dual-task interaction model emphasizes that a boost in activity occurs, AGREL emphasizes that the underlying neural structures (e.g., connection strengths) are being altered. This likely reflects a difference in the phenomenon that is the focus of investigation – memory for scenes or perceptual learning – and it is certainly possible that target detection results in both temporal selection and reinforcement learning. Attention and reward are closely related (Anderson et al., 2011), and their effects are difficult to disentangle (Maunsell, 2004). It will therefore be important to reconcile the AGREL and dual-task interaction models in the future.

Finally, additional research exploring the attentional boost effect in neuropsychological populations and in development could be invaluable for testing several claims of the dual-task interaction model. For example, examining whether the attentional boost effect is observed throughout the visual field in spatial neglect patients would provide a new test of how temporal selective attention and spatial selection interact (Robertson et al., 1998). Similarly, studying whether the attentional boost effect is present or impaired when the dopamine system is compromised, as in Parkinson’s disease and schizophrenia (Schultz, 1998), could shed light on the role of reinforcement learning in the effect. In addition, a recent account of autism suggests that it could reflect dysregulation of the LC-NE system (Mehler and Purpura, 2009). It is therefore possible that examining the attentional boost effect in this population could provide valuable insight into the nature of the attentional boost effect, as well as into the role of the LC-NE system in autism. Finally, because the enhancements that result from target detection are observable in memory only when demands on control processes are relatively low, it may also be useful to look at how changes in the development of multi-tasking ability and control (Luciana et al., 2005) influence the effect of target detection in behavioral and in brain activity.

## Conclusion

For decades research on attention and dual-task processing has been based on the notion that attention and cognition are limited in capacity, and research on these processes has consistently supported this claim. Recent data from the attentional boost effect, the target-mediated boost, and TIPL, however, suggest that there is more to attention than mitigating capacity limits in space. Rather, attending to a target can enhance the perceptual processing of concurrently presented information. Although not predicted by current theories of attention, these data can be accounted for by the proposal that temporal selective attention is broad in space, but selective in time. Additional research is needed to reconcile the dual-task interaction model with load theory’s claim that all perceptual resources are obligatorily used.

## Conflict of Interest Statement

The authors declare that the research was conducted in the absence of any commercial or financial relationships that could be construed as a potential conflict of interest.

## Footnotes

^1^Although targets are typically construed as items that lead to an overt or covert response, we define targets as items that lead to a change in planned behavior, including a no-go cue (cf. Makovski et al., 2012). These items require the updating of goal states and could therefore also lead to greater perceptual processing (e.g., Donchin and Coles, 1988; Aston-Jones and Cohen, 2005; Bouret and Sara, 2005; Zacks et al., 2007).

^2^For consistency, we refer to items that could be targets, but are not, as distractors. Distractors in RSVP tasks may have different effects on task performance than distractors that appear at the same time as a target in flanker tasks. Although they do not divert spatial attention from the target, distractors in RSVP tasks mask the target and could trigger inhibitory processes (e.g., Olivers and Meeter, 2008).

^3^A memory enhancement for scenes presented with targets has been referred to elsewhere as *fast task-irrelevant perceptual learning* (Leclercq and Seitz, 2012a,b,c).
